# Generation of retinal ganglion cells with functional axons from human induced pluripotent stem cells

**DOI:** 10.1038/srep08344

**Published:** 2015-02-10

**Authors:** Taku Tanaka, Tadashi Yokoi, Fuminobu Tamalu, Shu-Ichi Watanabe, Sachiko Nishina, Noriyuki Azuma

**Affiliations:** 1 Department of Ophthalmology and Laboratory for Visual Science, National Centre for Child Health and Development, Tokyo, Japan; 2 Department of Physiology, Faculty of Medicine, Saitama Medical University, Saitama, Japan

## Abstract

We generated self-induced retinal ganglion cells (RGCs) with functional axons from human induced pluripotent stem cells. After development of the optic vesicle from the induced stem cell embryoid body in three-dimensional culture, conversion to two-dimensional culture, achieved by supplementation with BDNF, resulted in differentiation of RGCs at a rate of nearly 90% as indicated by a marginal subregion of an extruded clump of cells, suggesting the formation of an optic vesicle. Axons extended radially from the margin of the clump. Induced RGCs expressed specific markers, such as Brn3b and Math5, as assessed using by quantitative PCR and immunohistochemistry. The long, prominent axons contained neurofilaments and tau and exhibited anterograde axonal transport and sodium-dependent action potentials. The ability to generate RGCs with functional axons uniformly and at a high rate may contribute to both basic and clinical science, including embryology, neurology, pathognomy, and treatment of various optic nerve diseases that threaten vision.

Retinal disorders are an important cause of blindness throughout the world^1^,^2^. Of the various types of retinal cells, photoreceptors and ganglion cells are primarily affected by retinal disorders; photoreceptors are generally affected by retinitis pigmentosa and retinal dystrophy^3^,^4^, while ganglion cells are generally affected by optic neuropathy and glaucoma^5^. Research aimed at achieving regeneration of the diseased retina has identified several candidate structures that contain precursors of key retinal cells, including the iris^6^,^7^, ciliary epithelium^8^, and Muller glial cells^9^,^10^. Two dimensional (2D) cell culture methods have been employed in this work; however, efficient regeneration of photoreceptors and the other retinal cells has not been achieved without application of exogenous factors that relate to retinal morphogenesis^11^,^12^,^13^, whereas self-induction by endogenous factors would have been ideal. The establishment of embryonic stem cell (ESC) and induced pluripotent stem cell (iPSC) technologies has recently allowed further advances in the generation of retinal cells.

The development of three-dimensional (3D) culture systems has led to more advanced work on differentiation of neural tissues from ESs and iPSCs, facilitating self-organisation of brain cortical tissue^14^, the pituitary gland^15^, and the retina starting from mouse and human ESCs^16^,^17^. First, the optic vesicle and continuous optic cup, which are two early developmental stages of the eyeball, were generated from ESC embryoid bodies. These tissues were then cut with scissors and continuously incubated in a floating culture system under a high-oxygen atmosphere, resulting in self-induction of almost all retinal layers^15^,^16^, including the ganglion cell and photoreceptor cell layers. In further advancements to this method, all layers of the murine retina were generated from ESCs and iPSCs, and retinal sheets were successfully transplanted into the subretinal space of mice with advanced retinal degeneration (rd1), which lacked the outer nuclear layer of the retina^18^.

Another series of 3D culture systems has been developed, enabling generation of the 3D retinal structure, including retinal ganglion cell and photoreceptor cell layers. In the primary experiment, adherent human embryonic stem cells and iPS cells were picked up mechanically and transferred to floating culture to be induced into an optic vesicle (OV)-like structure with retinal precursor characteristics. This structure was then reattached to a 2D culture dish, where it differentiated into the retinal cell lineage^19^,^20^. In subsequent experiments in 3D culture systems, the entire retinal layer, including the retinal ganglion cell layer and functional photoreceptors, was established from human iPSCs mainly using intrinsic cues^21^ and addition of small amounts of exogenous factors, as reported in previous studies^22^,^23^,^24^,^25^,^26^. In this experiment, an OV-like structure was directly differentiated into a retina without the process of optic cup formation. There is a critical difference between these two major methods; in the former, the OV-like structures were extruded from the embryoid body, while in the latter, aggregates of human iPSCs or ESCs were directly induced into OV-like structures. In both methods^17^,^21^, the entire retinal layer is regenerated, containing thick outer layers with well-differentiated photoreceptors and thin, immature inner layers comprised of RGCs. Moreover, the formation of RGC axons has not been realised using either 3D retinogenesis method.

The RGCs located in the ganglion cell layer of the retina typically have long axons, enabling them to connect the retina to various visual centres in the central nervous system, represented by the lateral geniculate body^27^. RGCs play roles in transmitting image-forming and non-image-forming visual information^28^, processed by photoreceptors, horizontal cells, amacrine cells, and bipolar cells, to higher visual centres via converging non-myelinated nerve fibres in the retina that continue into the myelinated optic nerve. Thus, the long axons of RGCs are crucial to transmitting visual images from the retina to the brain.

However, although culture of isolated RGCs with tiny axons from animal and human retinas has been achieved by immunopanning methods^29^,^30^,^31^, no study has reported the generation of RGCs exhibiting characteristic long, prominent axons in an *in vitro* culture system, with the exception of one report in which RGCs were developed from mouse iPSCs that carried an Atoh/Math5 lineage reporter^32^ and another report in which almost 30% of attached aggregates of RGCs derived from human iPSCs exhibited elongated axons with action potentials^33^.

Here, we describe a novel *in vitro* method that generated RGCs with long, functioning axons exhibiting action potentials and axonal transport, by combining cultivation of 3D floating aggregates with a subsequent 2D adhesion culture. This advance may facilitate both basic research on RGCs and translational research aimed at finding treatments for retinal diseases.

## Results

### Protocol for self-induction of RGCs from human iPSCs

**Figure 1:**
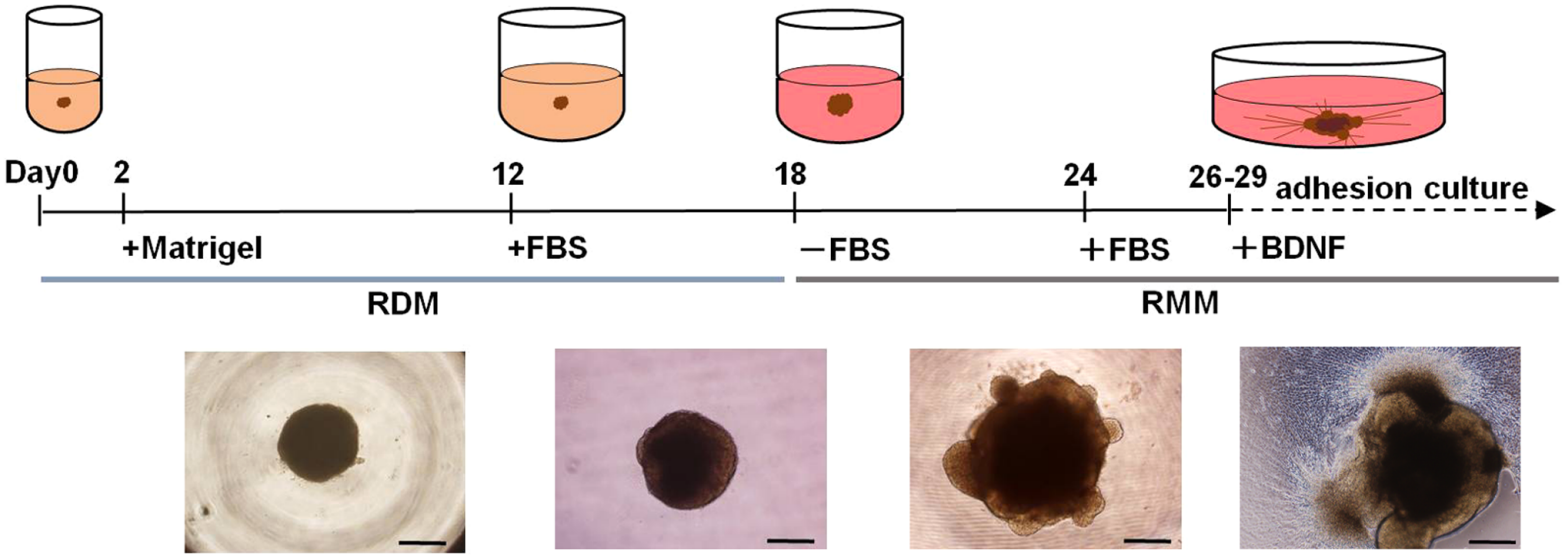
Schematic diagram of the protocol for self-induction of retinal ganglion cells. This protocol consisted of a period of suspension culture (3D) followed by a period of adhesive culture (2D) and resulted in observation of axonal elongation from retinal ganglion cells (RGCs) from human iPSCs starting within 30 days. Two basal media, retinal differentiation medium (RDM) and retinal maturation medium (RDM) were used. Significant morphological changes occurred after a medium change from RDM to serum-free RMM on D18, at which point optic vesicles (OVs) appeared to extrude from the cultured cell aggregates. Beginning with the start of adhesive culture on D26–29, axons grew out radially from the mass of new RGC bodies. Phase contrast micrographs were taken on D6, D18, D24, and D30 and are shown in sequence from left to right. Scale bar, 500 μm.

For purposes of generating RGCs, we modified a previously established^17^ 3D retinal regeneration protocol (Fig. 1). This method called for addition of both foetal bovine serum (FBS) and Matrigel to the culture medium; these components were not required by other protocols^20^,^21^, which also produced retinal cells efficiently. Therefore, we investigated the effects of FBS concentrations (1%–10%) on D12–18 and Matrigel concentrations (0.5%–2.0%) on D1–18. At all concentrations, embryoid bodies (EBs) were well organised, and OVs, which were maintained over the duration of the study, extruded from nearly 90% of the EBs (Table S1 and Fig. S1). In order to establish RGCs with long axons in a relatively short period, we changed the OV culture conditions from floating (3D) to dish (2D) to obtain cell attachment because the incipient ganglion cell layer was located on the surface of the OV. Initially, we severed the OVs from the EBs, which is predicted to lead to differentiation into neural retinal cells, as demonstrated previously^17^. However, maintenance of the cut OVs did not succeed, as documented in another report^34^. Therefore, we directly attached the OVs to the dish while still connected to the EBs, and the attached OVs were further differentiated into an extruded clump of cells on the dishes. Around 2 days after attachment, massive axonal growth from the margin of the clump was realised. The optimal timing of attachment was also evaluated. Attachment on D26–29 was ideal for elongation of axons from the marginal portion of the clump, which was thought to represent the RGC region (RGCR), while attachment prior to D24 or after D30 seemed suboptimal for efficient development of axons (Fig. S2). When retinoic acid was added 3 days prior to the attachment, axonal growth was promoted (Fig. S3); in contrast, if retinoic acid was continuously supplemented after adhesion, axons grew by around D32, but died back by around D40 (Fig. S4). Finally, following addition of BDNF (100 ng/mL) to promote neuronal survival beyond 3 days of retinoic acid administration, RGCs and their axons were stabilised until around D50 (Fig. S5).

### The time course of expression of transcription factors and protein markers associated with retinal development

**Figure 2:**
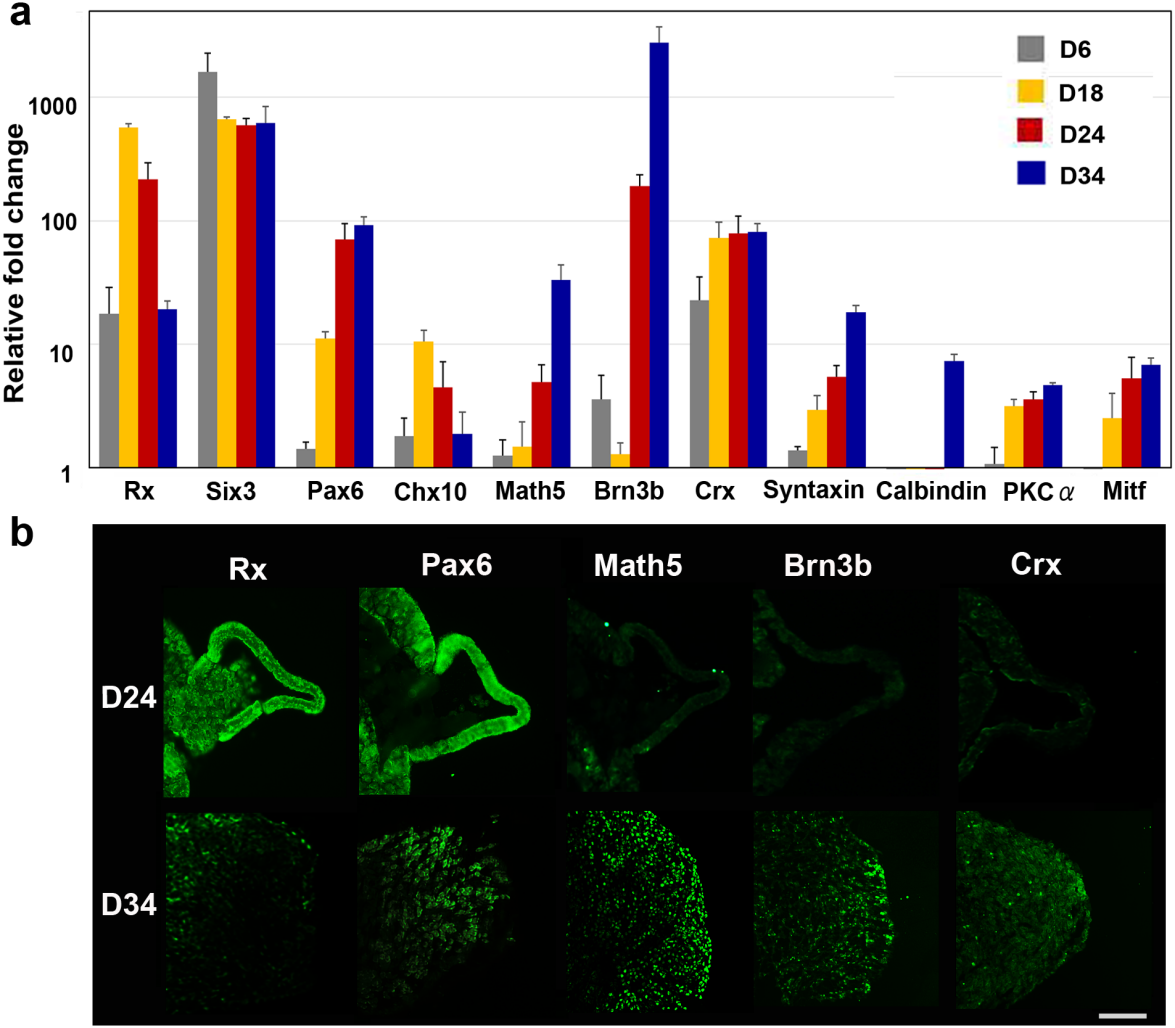
Commitment of human iPSCs toward a retinal lineage and generation of retinal ganglion cells (RGCs). (a) The time-dependent expression of transcription factors involved in retinogenesis was examined. Expression changes during differentiation of iPSCs to RGCs as revealed by quantitative PCR. Total RNA was extracted on D6, D18, D24, and D34. Retinal progenitor markers, including Rx, Six3, and Pax6, were apparently expressed until D18. Brn3b, the RGC-specific marker, was upregulated in a sporadic manner, following an increase in the expression of Math5, the upstream regulator of Brn3b. Crx, the marker of the cone-rod photoreceptor precursor, could also be identified starting at D18. Syntaxin, the amacrine cell marker, gradually increased following increased Brn3b expression. Expression levels of calbindin, a marker of horizontal cells, and PKCα, a marker of bipolar cells, were quite low. The expression of Mitf, a master controller of retinal pigment epithelium differentiation, was suppressed, indicating that these cells were in the neural retinal lineage. Expression levels of mRNAs were first normalised to the expression of HPRT1 and then to the expression on D0 (the start day of suspension culture), which was set at 1.0. Error bars represent means ± SDs of the samples from three independent experiments. The vertical axis is a base-two logarithmic scale. (b) Immunohistochemistry for Rx, Pax6, Brn3b, Math5, and Crx in the optic vesicle (OV) on the floating EB on D24 and in extrusions of cell clumps that differentiated from the OVs after attachment of the EBs on D34, using each serial section. Rx and Pax6 were expressed in the OV and the extruded clump of cells. Positive immunostaining for Brn3b and Math5, the most specific markers for RGCs, was observed not in the OV, but was observed in the clump and was localised to the outer margin of the clump. Crx, a marker for primitive photoreceptors, was not expressed in the OV but was faintly expressed in the clump. Scale bar, 100 μm.

The OV is derived from the eye field in the forebrain, a process in which several transcription factors, called eye-field transcription factors, play principal roles^35^. The retinal homeobox gene, Rx, and the paired box gene 6, Pax6, which belong to this group of eye-field transcription factors, are crucial in early retinogenesis^36^. During the period of attachment of the cultured EB with its extruded OV to a dish, temporal gene expression analysis clearly revealed that upregulation of Rx began on D6, when the EB had maturely formed, and peaked on D18, when the OVs began to develop. This was followed by upregulation of its downstream target Pax6. Math5, the bHLH transcription factor, is expressed in post-mitotic retinal precursor cells under direct control by Pax6^37^. Math5 crucially regulates RGC formation, and Math5 mutant mice lack RGCs^38^,^39^. In humans, Math5 mutation results in optic nerve aplasia^40^. Brn3b, known as the most reliable RGC marker, is a member of the POU-domain family of transcription factors, and its expression is controlled by Math5^41^. Brn3b is believed to determine RGC fate by acting as a repressor of differentiation to non-RGC cell types^42^, a process in which Islet1, an LIM-homeodomain factor, acts as a coregulator^43^. In our experiments, expression of Brn3b was relatively low until D18 and then dramatically increased until D24, when the OVs were well developed from the floating EBs following an increase in Math5 expression. Chx10 is associated with the proliferation of retinal progenitor cells. It is initially expressed in all retinal progenitors, but later is restricted to bipolar cells^44^. In our experiments, expression of Chx10 became apparent at D18 and was maintained until at least D34, when RGCs and axons developed after attachment of the EB. Crx, a cone-rod-containing gene, shows restricted expression in premature and mature cells of photoreceptor lineage^45^,^46^. Crx expression was also first observed at D18, following Chx10 expression. Amacrine cells start to differentiate immediately after RGC differentiation^47^. In our experiments, syntaxin, an amacrine cell marker^48^,^49^, showed delayed expression following Brn3b expression. The markers associated with horizontal and bipolar cells, both of which develop at later stages of retinogenesis, showed little expression until D34. The time course of gene expression was quite consistent with that in human retinogenesis. Mitf, a microphthalmia-associated transcription factor and the master regulator of melanocyte and retinal pigment epithelium (RPE) development^50^, was not highly expressed in the present study, suggesting that our induction protocol induced human iPSCs into the neural retinal lineage but not into the RPE lineage (Fig. 2a).

Immunohistochemistry also showed expression of Rx and Pax6 in both OVs and the clump, while expression of Brn3b and Math5 was observed only in the clump, localised within the margins of the clump. In contrast, low expression of Crx was observed within the clump (Fig. 2b). Calbindin and PKCα were not detected in either the OV or the clump (Fig. S6).

### Structure of human iPSC-derived RGCs by light and electron microscopy

**Figure 3:**
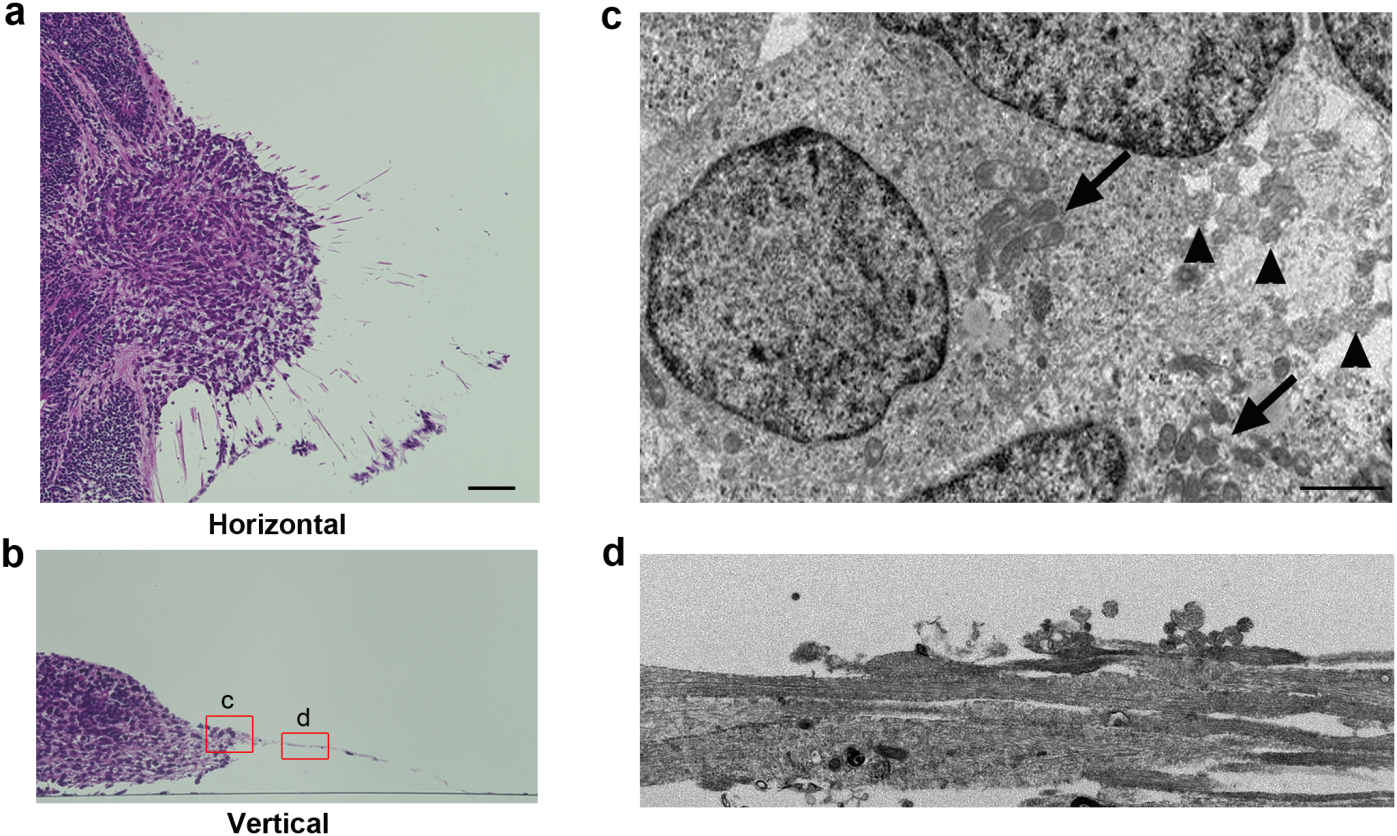
Light and electron microscopy of induced retinal ganglion cells and their axons. Horizontal (a) and vertical (b) sections stained with haematoxylin and eosin showed that axons radiated from the outer margin of the clump of cells corresponding to the optic vesicle (OV), which extruded from the embryoid body late in 3D culture. Somata of new retinal ganglion cells (RGCs) were present in the marginal portion of the extruded clump and showed variations in size and relatively large nuclei, some of which has Nissl's body characteristics of RGCs, while the main part of the embryoid body (MBEB) contained many irregular rosettes. (c) Electron microscopy analysis of the square portion (c) in panel (b) revealed that cell bodies of RGCs contained prominent rough endoplasmic reticulum (arrows) and that axons (arrowheads) developed from a portion of the axon hillock. (d) Electron microscopy analysis of the square portion (d) in panel (b) revealed straight axons of varied calibre, ranging in diameter from 1.7 to 2.2 μm and lacking myelination. The axons contained numerous neurotubules. Scale bars, 80 μm in panels (a) and (b); 5 μm in panels (c) and (d).

We investigated the structure of the RGCs by light and electron microscopy. After the OV became attached around D26–29, it became complanate, filled with cells, and showed radiation of numerous axons from the outer margin at the dish surface. The cells in the clump were apparently different from those in the main body of the EB, which was filled with small round cells. The cells in the clump showed relatively large cell bodies (range: 16.1–23.6 μm; average: 21.6 μm, n = 10) with clear cytoplasms and nuclei and spindle-shaped or round cell bodies, sometimes accompanied by a prominent axon. These findings were identical to those of RGCs *in vivo*, whose cell body sizes ranged from 12 to 25 μm^51^. Notably, several cells located peripheral to the structure had Nissl bodies (Fig. S7), which is considered characteristic of RGCs^52^. However, Nissl bodies were not detected in other types of cells, including those in rosettes. The axons were eosinophilic, had few branches, and grew radially from the clump (Fig. 3a, b).

Electron microscopy of D35 specimens demonstrated that the RGCs, which had axons, seemed to be relatively large (range: 16.0–24.1 μm; average: 20.6 μm, n = 10) and were stratified at the margin of the clump. These RGCs contained prominent rough endoplasmic reticulum, which represented the Nissl bodies that were observed by light microscopy. The axons developed from the axon hillock, which exhibited a characteristic conical cell body with neurotubules but no rough endoplasmic reticulum (Fig. 3c), which have also been detected in RGCs *in vivo*^53^,^54^ and define the end of the soma of neurons. The axons contained numerous neurotubules. The diameters of the axons varied (range: 1.7–2.2 μm; average: 1.8 μm, n = 10), and the axons were not myelinated (Fig. 3d). These findings were identical to those of axons in the retinal nerve fibre layer and in the optic nerve anterior to the lamina cribrosa, whose diameters range from 0.6 to 2.0 μm^51^.

### Determination of RGC lineages

**Figure 4:**
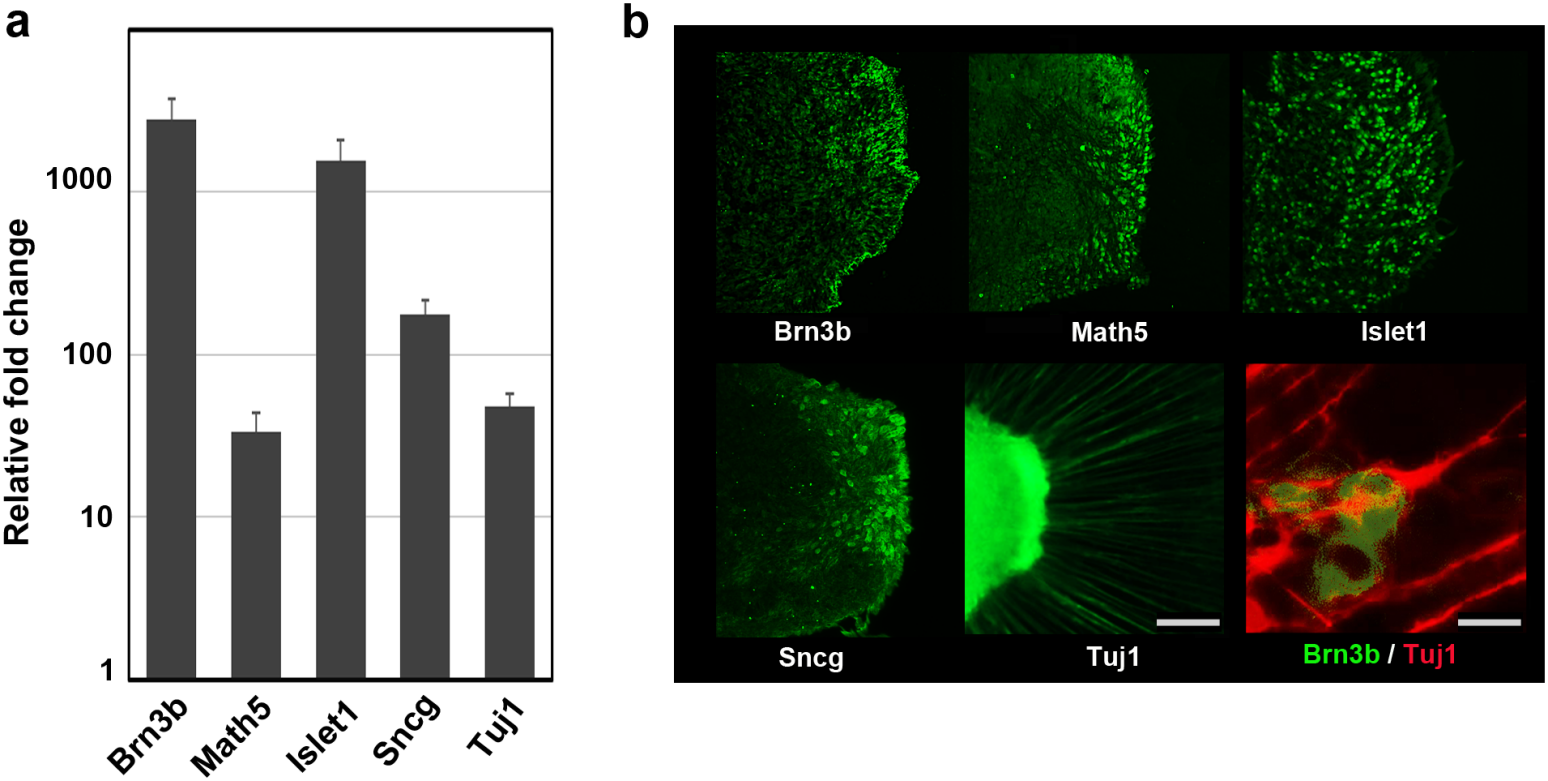
Expression of characteristic markers related to retinal ganglion cells. The expression levels of representative retinal ganglion cell (RGC) markers, Brn3b, Math5, Islet1, γ-synuclein (Sncg), and β3-tubulin (Tuj1), were examined. (a) Quantitative PCR analysis of total RNA extracted on D34 was used. All markers associated with the RGC lineage were obviously upregulated more than 30-fold. In particular, the mRNA levels of Brn3b and Islet1 were increased dramatically. The mRNA expression levels were first normalised to HPRT1 expression and then to mRNA expression on D0. Error bars represent means ± SDs of three samples. The vertical axis was a base-two logarithmic scale. (b) Immunohistochemistry for Brn3b, Math5, Islet1, Sncg, and Tuj1 on D34. Whole mount staining for Tuj1 and serial section staining for the other targets were analysed. Positive immunostaining for Brn3b and Math5, the most specific markers for RGCs, was localised to the outer margin of the extrusion that differentiated from the optic vesicle. Sncg, another marker for RGCs, also showed positive staining in the marginal portion of the clump. Islet1, a marker for RGCs and amacrine cells, was expressed in the inner part of the clump. Axons were strongly positive for Tuj1, and the axons were confirmed to grow from Brn3b-positive cells. Scale bar, 100 μm. Scale bar for the double staining of Brn3b and Tuj1, 10 μm.

To confirm that the cells with axons in the margin of the clump differentiated from the OV were RGCs, we examined the expression and distribution of typical markers associated with RGCs. Real-time PCR revealed a more than 30-fold increase in the expression of Brn3b, Math5, Islet1, γ-synuclein (Sncg), and Tuj1. Brn3b, the most specific marker in this group, showed a nearly 3,000-fold increase in expression at D34 compared with D0 (Fig. 4a). Immunohistochemistry showed positive staining for all markers except Tuj1 in the marginal portion of the clump, which was therefore thought to be the incipient RGC layer. Math5, an upstream transcription factor for Brn3b, and Sncg, another RGC marker and contributor to neurofilament integration^55^, showed similar distributions in the marginal portion of the clump. Islet1, a marker of RGCs and amacrine cells, was positive both in the margin and further toward the interior of the clump, suggesting that amacrine cells also developed there. Notably, Brn3b-positive cells apparently localised to the outermost margin of the clump at the surface of the dish, which strongly indicated that the margin was the RGCR and that the long radial axons originated from the RGCR. The costaining of Brn3b and Tuj1 demonstrated that the axons originated from the Brn3b-positive cells (Fig. 4b).

### Sectional gene expression associated with RGCs and other retinal cells

**Figure 5:**
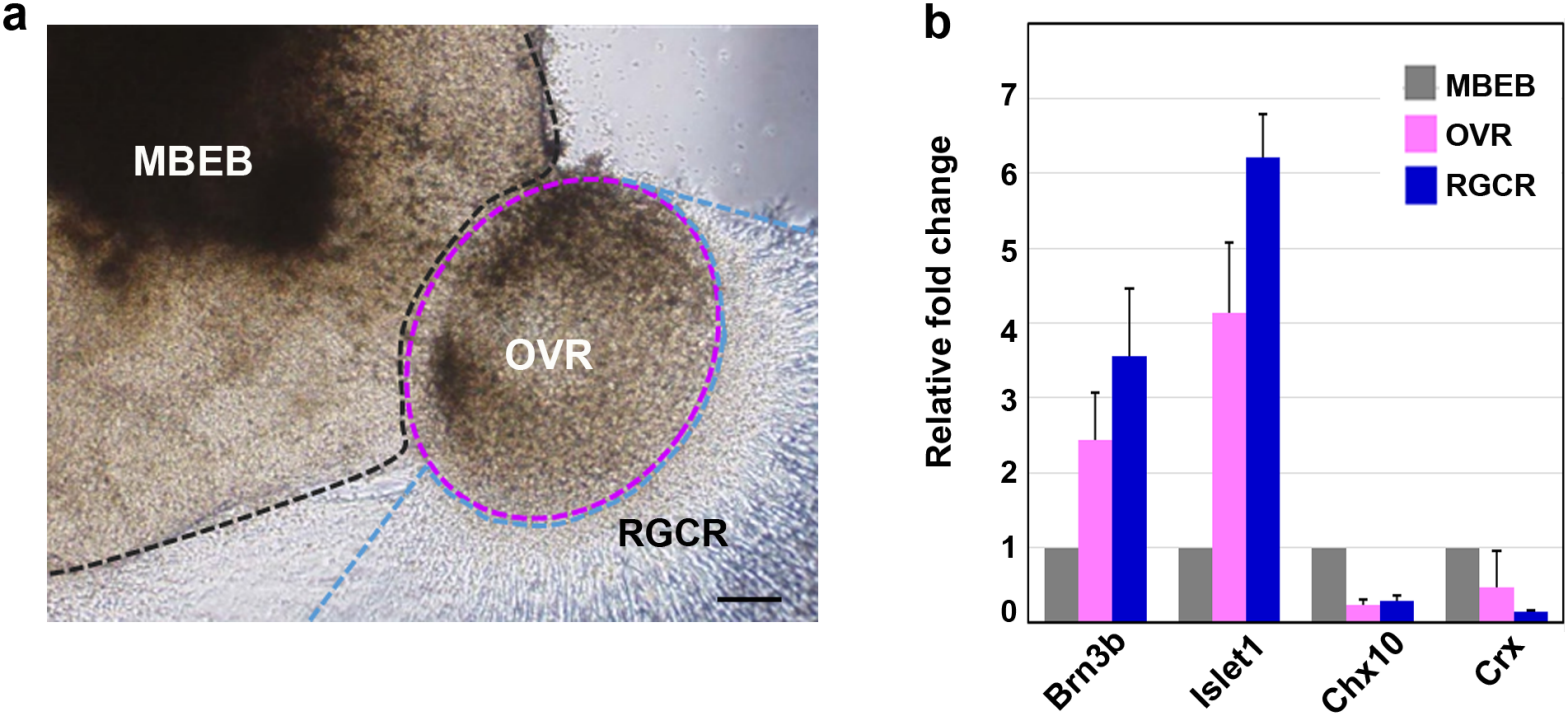
Regional differentiation of retinal ganglion cells detected by RT-PCR. (a) Each colony on D34 was mechanically divided into three regions, namely the main body of the embryoid body (MBEB), the extruded clump of cells differentiated from the optic vesicle except for its marginal part (OVR; optic vesicle region), and the retinal ganglion cell region (RGCR). The latter was comprised of the marginal layer of the extrusion and the axons. (b) The gene expression of neural retinal markers in each region was investigated by quantitative PCR analysis. Analysed markers included Brn3b (RGCs), Islet1 (RGCs, amacrine cells), Crx (photoreceptors), and Chx10 (neural retina progenitor and bipolar cells). The RGCR showed high expression of Brn3b compared with the other two regions, but lacked Crx expression, suggesting that the RGCR was specifically differentiated into the RGC lineage. The mRNA expression levels were first normalised to HPRT1 expression and then to mRNA expression levels on D0. Error bars represent means ± SDs of three samples from three independent experiments. Scale bar, 100 μm.

For further confirmation of the presence of localised RGCs in the supposed RGCR, we mechanically divided the adherent structural complex into three parts: the RGCR, OVR, and MBEB (Fig. 5a). Notably, Brn3b showed nearly four-fold higher expression in the RGCR than that in the MBEB. In addition, Crx expression was strongly suppressed in the RGCR. These results obviously demonstrated that the RGCR was highly specified into the ganglion cell lineage. Islet1 (a marker for ganglion and amacrine cells) was also up regulated in both the RGCR and OVR, which was consistent with the distribution of Islet1 expression confirmed by immunostaining (Fig. 5b). We also noticed that the isolated RGCR survived for approximately 10 days after separation from the OVR, which suggests that mechanical purification of RGCs is possible after growth of abundant axons.

### RGC axon-related protein expression and determination of types of neurofilament proteins in early retinal gangliogenesis

**Figure 6:**
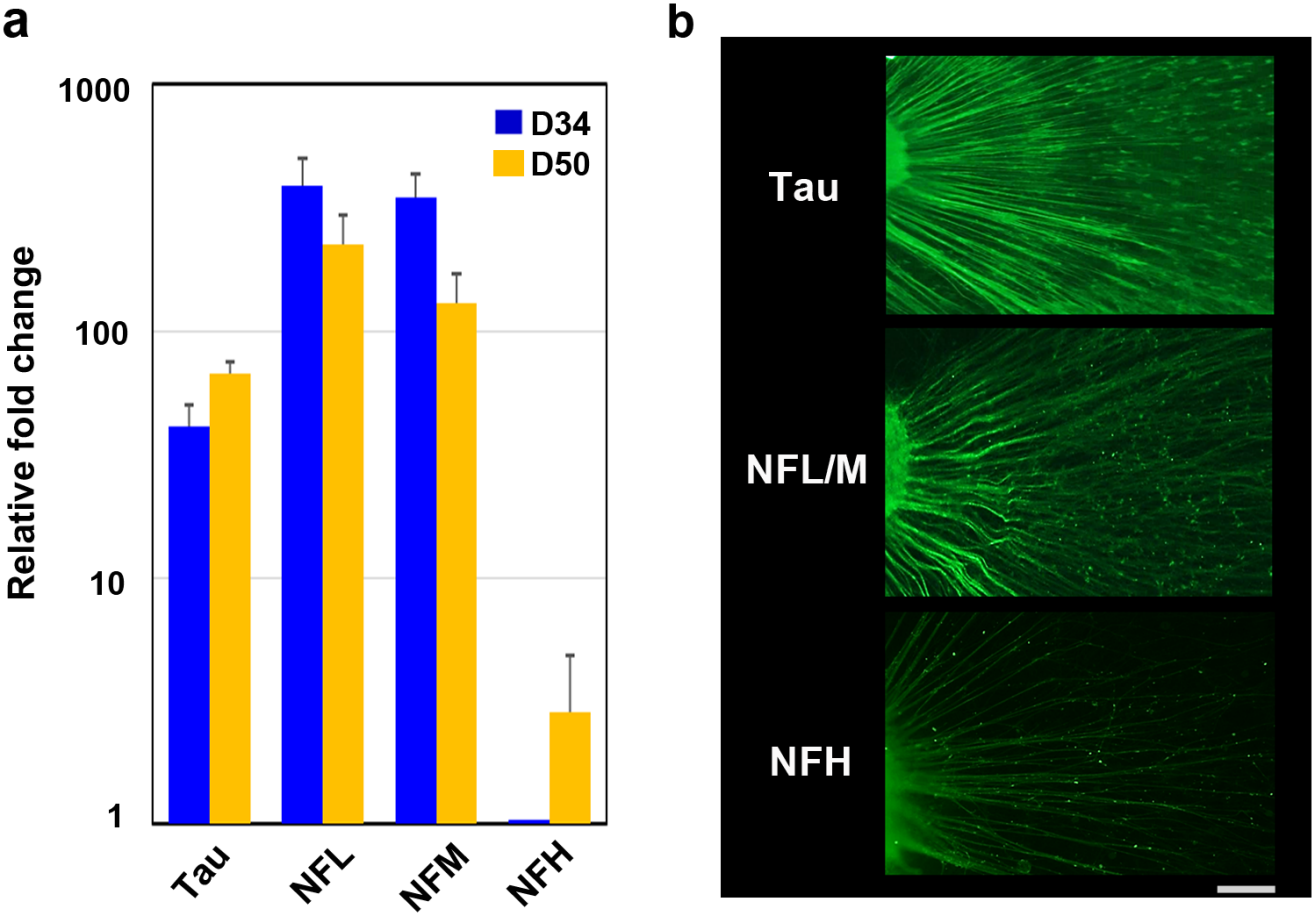
Cytoskeleton in axons elongated from retinal ganglion cells. (a) Quantitative PCR analysis was applied to investigate nerve fibre markers, including neurofilament (NF) light (NFL), NF middle (NFM), NF heavy (NFH), and tau, using total RNA extracted on D34 and D50. The expression of each marker, including NFL, NFM, and tau, was already high at D34, while that for NFH was increased on D50 compared with D34, probably a reflection of axonal maturation. (b) Immunohistochemistry clearly showed positive staining in all axons for NFL, NFM, and tau at D34 and D50 and for NFH at D50. The mRNA expression levels were first normalised to HPRT1 expression and then to the mRNA expression levels of NFH on D34. Scale bar, 100 μm.

To confirm that the apparent axons emanating from the RGCR resembled typical axons, we used tau and neurofilament (NF) immunostaining (Fig. 6b). The time-series expression of all NF components—neurofilament-light (NFL), neurofilament-medium (NFM), and neurofilament-heavy (NFH)—was studied in parallel (Fig. 6a). Microtubules, NFs, microtubule-associated proteins (MAPs), and actin play crucial roles in axonal development^56^. Tau, which is a MAP, contributes to polymerisation, stabilisation, and organisation of microtubules and promotes axonal growth and effective axonal transport^57^. Once tau is hyperphosphorylated, the axonal transport process and cell survival of RGCs are compromised^58^. The axons detected in our study were clearly stained for tau and associated with evidence of axonal transport. NFs are the intermediate filaments of neurons and are especially abundant in CNS axons, where they occur as heteropolymers made up of four subunits, namely NFL, NFM, NFH, and α-internexin^59^. NFs have essential roles in radial axonal growth and maintenance of axon calibre, therefore mediating the ability to transmit action potentials^60^. Both NFL and NFM appear early in retinogenesis, while synthesis of NFH is delayed^61^. In our experiments, NFL and NFM were already highly expressed at D34 and expression continued until D50, while expression of NFH was quite low at D34 and gradually increased until D50. Specimens from D34 showed positive staining for NFL/M but weak staining for NFH, in agreement with the expression time series study. We observed temporally varying gene expression not only of transcription factors but also of genes associated with structural components of RGCs.

### Axonal transport in human iPSC-derived RGCs

**Figure 7:**
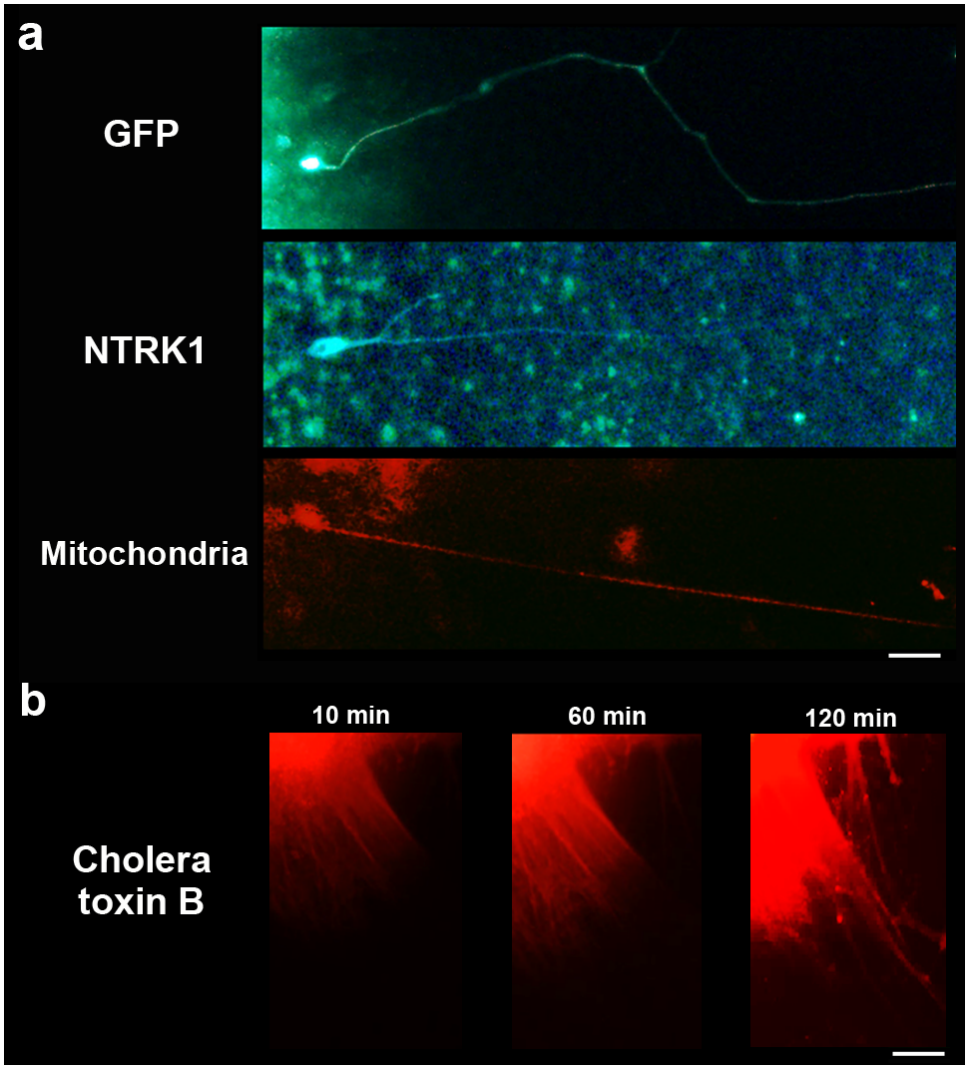
Axonal transport in retinal ganglion cells. (a) Axonal transport was investigated for investigation of anterograde rapid flow employing NTRK1 and mitochondrial expression vectors. GFP was also used as a control. Plasmid vectors that carried each of these substances as well as the CMV promoter were directly introduced into the cell body using electroporation around D34. At approximately 7–10 h post-introduction, GFP expression was observed, which indicated that effective introduction of vectors by electroporation had been obtained. NTRK1 and mitochondria were identified in the cell body and in the axon, the latter location indicating anterograde rapid axonal transport. Scale bar, 80 μm. (b) A time series of axonal transport was also confirmed by the injection of Alexa-Fluo-555 conjugated cholera toxin B into retinal ganglion cell region. Cholera toxin was transported from the cell body to the peripheral area of axons by anterograde flow within approximately 2 h after injection. Scale bar, 100 μm.

To establish axonal function, we tested for axonal transport in the apparent axons that radiated from the RGCR. Axonal transport showed two characteristic flows, slow and fast, both of which had anterograde and retrograde components. RGCs and their axons are known to be rich in mitochondria and to maintain homeostasis by axonal transport, as in other neurons. Neurotrophic tyrosine kinase receptor 1 (NTRK1), a member of the neurotrophic tyrosine kinase receptor family, is the receptor for nerve growth factor and is expressed in RGCs, especially when the cells are damaged^62^,^63^,^64^. Using NTRK1 and mitochondria as tracers, we directly introduced plasmid vectors composed of GFP-NTRK1 with a CMV promoter and mCherry-mitochondria with a CMV promoter into the cell body of RGCs around D34 by electroporation to analyse anterograde rapid axonal transport. Several axons demonstrated anterograde axonal transport after approximately 7–10 h post-introduction. The cell bodies corresponding to these axons expressed these factors as well (Fig. 7a).

To detect a time series of axonal transport, Alexa-Fluo-555 conjugated cholera toxin B, which is also known as a tracer of axonal transport^58^, was administrated. Slow and fast anterograde axonal flow were clearly observed by injection of the toxin into RGCR (Fig. 7b, Fig. S8, Supplemental Video 1). The transport was blocked by an addition of 1 mM colchicine (Fig. S9, Supplemental Video S2 and S3).

### Human iPS cell-derived RGCs showed neuronal excitability

**Figure 8:**
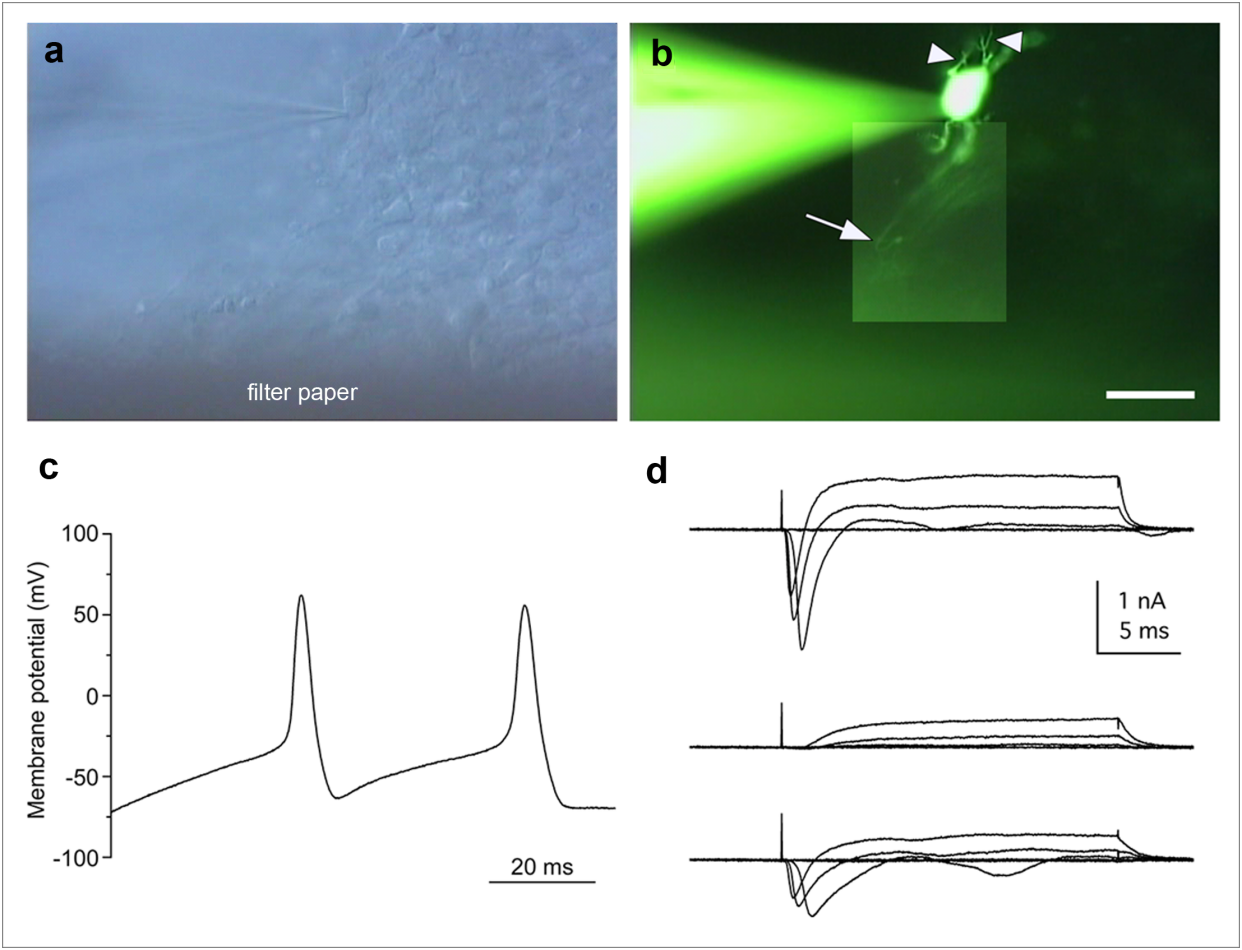
Action potentials of the retinal ganglion cells. (a) Slice preparation of the cultured colony with recording electrode under DIC optics. (b) Composite photograph of an iPS cell-derived retinal ganglion cell filled with LY under fluorescence illumination. This cell exhibited some dendritic processes (arrowheads) and an axonal process (arrow). The same field is shown in (a) and (b). (c) Whole-cell recording of the retinal ganglion cells revealed action potentials in current-clamp mode (current injection of 60 pA). (d) A family of currents was recorded in voltage clamp mode in response to depolarising steps from a holding potential of −71 mV to target voltages increasing from −41 to +19 mV in 20-mV increments (upper traces). The fast inward currents were blocked by TTX (middle traces) and were recovered by washing (lower traces). Action potentials and inward currents were recorded from the LY-labelled cell shown in panel (b). Scale bar in panel (b), 30 μm.

To determine whether iPSC-derived RGCs could generate action potentials, we examined the RGCs in the RGCR (Fig. 8a). The RGCs each had a long axon process extending to the filter paper side and possessed some dendritic processes (Fig. 8b). Electrophysiological analysis of cells with an axon process revealed repetitive action potentials in response to current injection through the recording pipette in current clamp mode (4 of 5 cells; Fig. 8c). The resting membrane potential and the amplitude of the first action potential were −81.8 ± 26.8 mV and 56.8 ± 24.6 mV, respectively (n = 4). The cells that generated action potentials exhibited tetrodotoxin (TTX)-sensitive sodium-dependent currents with a maximum amplitude of 1,248 ± 573 pA (n = 4), followed by outward currents (Fig. 8d).

## Discussion

In this study, we generated RGCs with functioning axons from human iPSCs. The present quantitative PCR and immunohistochemistry results showed a gene expression profile and protein markers characteristic of RGCs, including Brn3b, Math5, Islet1, Sncg, and Tuj1. RGCs induced from ESCs and iPSCs in previous studies carried markers characteristic of RGCs^17^,^21^,^65^, but were not accompanied by long axons. RGCs have also been generated in 3D culture systems, but without observation of the axons that would be expected with formation of the nerve fibre layers of the retina and the nerve fibre bundle of the optic nerve. In contrast, RGCs observed in the present study had long, prominent axons expressing NFs, Tuj1, and tau and growing in straight lines on the culture plate. The functionality of the axons was demonstrated by observation of axonal flow and action potentials. Anterograde axonal transport was identified by induction of both NTRK1- and mitochondria-specific vital stains and cholera toxin B into cell bodies. Sodium-dependent action potentials were demonstrated by patch clamp techniques.

RGCs were generated mostly in an OV-like clump of cells extruded from an EB where the layers of neural retina were supposed to differentiate. Most RGCs were localised to the marginal portion of the clump that adhered to the bottom of the culture dish. Because RGCs in vertebrates are located in the most anterior layer of the retina, which coincides with the marginal portion of the OV and optic cup in both embryogenesis and in previously established 3D culture systems, it is not surprising that our RGCs differentiated specifically at the surface of the clump, the inner portion of which may be an agglomerate of other retinal cell types. Relatively large cells with prominent nuclei, consistent with RGC morphology, were stratified at the marginal portion of the clump and were positive for Brn3b, the most specific marker for RGCs. Sectional analysis of RNA expression also confirmed that Brn3b expression was highest at the marginal portion of the clump without accompanying Crx expression, implying that the marginal portion of the clump had been specified purely into RGCs. This marginal portion, the RGCR, was able to survive for several weeks separated from the parent clump of cells (Fig. S3), suggesting the possibility of mechanically purifying RGCs for use in further investigations. Retinal cell determination progresses via a well-organised spatio-temporal pattern, in which RGCs appear initially and are followed by amacrine cells, photoreceptors, horizontal cells, bipolar cells, and Muller cells, a process that is highly conserved among species^47^,^66^In monkeys, RGC development begins around D25^67^, and Brn3b, a characteristic transcriptional factor, is expressed in the retina of the human foetus at least by 8 weeks of gestation^68^. Thus, the timing of the marker expression and development of RGCs in our experiment resembles the process of the genesis of RGCs *in vivo*.

The axonal growth pattern differs significantly between 2D (on-dish) and 3D cultures. Two-dimensional culture methods for CNS neurons, including hippocampal^69^, cortical^70^,^71^, and hypothalamic cells^72^,^73^, have been well established. These primary cultures can be augmented by coating the bottom of the culture dish with extracellular matrix components, including laminin, fibronectin, cadherin, and collagen, through which axons can extend^74^. In addition, neuroprotective agents, including BDNF^75^,^76^, nerve growth factor^77^, and CTNF^78^,^79^, can easily be provided to the cells uniformly by adding them to the culture medium to further support neuron and axonal growth.

In contrast, the 3D culture approach has recently achieved *in vitro* organogenesis of CNS structures^80^,^81^, including the retina^16^,^17^,^34^, the pituitary gland^15^ and heterogeneous neural tissue^82^, in which axons generally extend into an extracellular matrix gel that contains collagen, fibronectin, or cadherin. Although 3D-cultured neurons seem more suitable for investigating stratified structures, branching, and synaptogenesis, the formation of long axons has not been obtained by previous 3D culture methods for retinogenesis. Achieving differentiation to normal RGCs by these methods may be too challenging because the surface of the self-induced retina directly contacts homogeneous culture fluid, which contains no extracellular matrix geometries capable of providing a foothold for horizontal extension of axons from RGCs. Expecting to achieve targeted horizontal extension of axons along the surface of the retina towards the optic nerve head, probably guided by extracellular signals such as Shh, BMP, and BDNF^83^, would be even more difficult because self-induced retinas lack both the optic stalk and the nerve head.

Even in the environment of 2D culture where concentrations of multiple growth factors can be reliably controlled, neither self-induction of retinal layers nor prominent outgrowth of axons from RGCs have been achieved thus far. In the present study, axons extended from RGC bodies in response to a switch of the culture method from 3D to 2D at an optimal time, when the polarity and location of the new RGCs had just been determined. Supplementation with BDNF, a protective agent for RGCs, further supported axon growth. These spatio-temporal steps may be requirements for axon growth from RGCs. However, development of axons by a purely 3D culture method merits further research. RGC axons grown by the 3D to 2D culture method we established were formed uniformly, at a high rate, and in a short time and may contribute to various endeavours in basic and clinical science on human RGCs. For example, embryogenesis, nerve path finding, synaptogenesis, and interactions between nerve and glial cells in coculture may be easily investigated *in vitro*.

Our RGCs, which possessed long axons, also provided an excellent tool for investigating the pathogenesis and pathophysiology of optic nerve diseases. The optic nerve is composed of some 800,000–1,000,000 RGC axons^51^ and can be anterogradely or retrogradely damaged by various crises, including ischemia, infection, trauma, inflammation, tumours, and genetic disorders. These can result in cell death and clinically severe visual impairment or blindness, such as glaucoma. Glaucoma, a disease caused by high intraocular pressure, is an important cause of blindness globally. The pathogenesis and pathophysiology of many diseases associated with RGCs remain to be elucidated because of the difficulty of clinical examination of the deepest portions of the eyeball. RGCs induced by iPSCs derived from patients with RGC-associated diseases, especially genetic diseases, may further our understanding of the underlying molecular mechanisms of pathogenesis and pathophysiology. Precedents for this are already documented for other neurodegenerative diseases, including Parkinson' disease and Alzheimer's disease^84^,^85^. Moreover, the efficacy and toxicity of drugs for neuroprotection and support of axonal outgrowth and regeneration relevant to human RGC-associated diseases could be examined *in vitro* with our system with far more ease, accuracy, and safety than in the era of animal models. Indeed, an *in vitro* clinical study relevant to cardiac disease has already started^86^,^87^. In addition, future therapies involving isolation of human RGCs for transplantation to repair damaged optic nerves may benefit from our method, though several issues remain to be elucidated, including how to induce complex axonal projection patterns and synaptogenesis in the CNS target areas.

## Methods

### Ethical statement

The Ethics Committee of the National Institute for Child and Health Development (NCCHD) approved all experimental protocols (approval number #686, July 3, 2013). All experiments handling human cells and tissues were carried out in accordance with the Tenets of the Declaration of Helsinki.

### Human iPSC culture

Human iPSCs (HPS0007_409B2 cell passage 29) were obtained from RIKEN BRC (Japan) and were maintained on a feeder layer of mouse embryonic fibroblasts (MEFs) inactivated by irradiation in Primate ES medium (ReproCELL) supplemented with 10 ng/mL recombinant human basic fibroblast growth factor (bFGF; Invitrogen). For passaging, hiPSC colonies were detached and recovered from the feeder layer by treatment with dissociation solution (ReproCELL) at 37°C for 8 min. The detached hiPSC clumps were broken into smaller pieces consisting of several tens of cells by gentle pipetting. Cell passaging was performed at a 1:3 to 1:4 split ratio.

### Induction of differentiation to RGCs

The induction of hiPSC differentiation employed a procedure based on SFEB methods^17^. hiPSCs were dissociated to single cells in TrypLE Express (Invitrogen) containing DNase I (0.05 mg/mL; Roche) and Y-27632 (10 μM; Wako) and were resuspended in retinal differentiation medium (RDM; G-MEM supplemented with 20% KSR, 0.1 mM nonessential amino acids, 1 mM pyruvate, 0.1 mM 2-mercaptoethanol, 100 U/mL penicillin, and 100 μg/mL streptomycin) containing Y-27636 and IWR-1e (20 and 3 μM, respectively; Merck Millipore). After separation from the feeder cells by decantation (the feeder cells adhered to the gelatine-coated bottom of the dish), the floating hiPSCs collected from the medium were seeded into V-bottomed low-cell-adhesion 96-well plates (Sumitomo Bakelite) at 9,000 cells per well. Matrigel (growth factor-reduced; BD Bioscience) was added to a final concentration of 0.5%–2% in the medium on day 2 (the day the suspension culture was initiated was defined as D0). On D12, the aggregates were transferred to low-cell-adhesion 24-well plates, and the medium was replaced with RDM containing Matrigel (0.5%–2%) and FBS (1%–10%). On D15, CHIR99021 (3 μM; Wako) and SAG (100 nM; Enzo Life Science) were added to the medium, and the suspension culture was continued for another 3 days. On D18, the aggregates were transferred to retinal maturation medium (RMM; DMEM/F12-Glutamax medium containing the N2 supplement, 100 U/mL penicillin, and 100 μg/mL streptomycin) and were then cultured in the absence of FBS. The adhesion culture started when the aggregates were transferred to poly-d-lysine/laminin-coated 24-well plates (BD Bioscience) in RMN medium containing FBS and 100 ng/mL BDNF (R&D Systems). The start day varied from D26 to D29, and the addition of retinoic acid (0.5 μM; Sigma) 3 days prior to the start of adhesion culture was preferred. The concentration of FBS was increased stepwise from 1% up to 10% over the course of the adhesion culture.

### Real-time reverse transcription polymerase chain reaction (RT-PCR)

Total RNA was extracted from cells using an RNeasy Mini Kit (QIAGEN). The expression levels of the mRNAs in each RNA sample were determined using the StepONE Sequence Detection System (Applied Biosystems). RT-PCR was performed using a One Step SYBR PrimeScript PLUS RT-PCR Kit (TaKaRa Bio). The primers used in this study are listed in Supplementary Table 1. The thermocycler conditions were as follows: an initial hold at 42°C for 5 min; incubation at 95°C for 10 s; and then 40 cycles at 95°C for 5 s and 60°C for 31 s. The expression of mRNA was assessed by evaluating threshold cycle (C_T_) values. The C_T_ values were normalised to the expression level of hypoxanthine phosphoribosyltransferase 1 (HPRT1), and the relative amount of mRNA specific to each of the target genes was calculated using the 2^−ΔΔCT^ method.

To perform sectional gene expression analysis, adherent tissues of EBs and extruding OVs were defined as three parts: the RGCR, which was located at the outermost 2–3 layers of cells in the clump of cells; the OVR, which was an adherent structure of OVs excluding the RGCR; and the MBEB, which was an adherent structure of the EB. The regions were obviously identified under a SZ61 stereomicroscope (Olympus), and mechanical separation using a 25-G needle was performed under the same microscope.

### Frozen section preparation

Specimens were fixed in 4% paraformaldehyde in 100 mM phosphate buffer for 3 h at 4°C, then rinsed in phosphate buffer, osmotic pressure-conditioned in a graded series of sucrose solutions in phosphate buffer up to 30%, and embedded in OCT compound (Tissue-Tek; Sakura Finetek). Each block was serially sectioned at 5-μm thickness using a CryoStar NX70 (Thermo Fisher Scientific).

### Immunohistochemistry

Immunostaining was performed using frozen sections or whole cells fixed on the dish. Whole cells specimens were fixed with 4% paraformaldehyde (pH 7.0) for 20 min at room temperature. After two rinses with PBS, specimens were incubated with 0.1% Triton X-100 for 15 min at room temperature and then washed three times with PBS for 5 min each. Specimens of frozen sections or whole cells were then incubated with 3% bovine serum albumin (BSA) for 30 min at room temperature followed by primary antibody incubation for 16 h at 4°C. The primary antibodies used in this study and their dilutions are listed in Supplementary Table 2. Secondary antibody reactions were carried out by incubation with the corresponding species-specific Alexa Fluor-488-conjugated antibodies (1:500, Invitrogen) for 1 h at room temperature in the dark. After four washes with PBS for 5 min each, specimens were mounted with ProLong Gold Antifade Reagent with DAPI (Invitrogen) and viewed with an IX71 inverted research microscope (Olympus) or BZ-9000E (KEYENCE).

### Haematoxylin and eosin staining

Specimens were fixed in 4% paraformaldehyde in 100 mM phosphate buffer for 3 h at 4°C, rinsed in water, dehydrated in a graded series of alcohols/xylene, and embedded in paraffin. Each block was serially sectioned at a 3-μm thickness. Deparaffinized sections were then stained with haematoxylin and eosin.

### Transmission electron microscopy

Specimens were fixed in 2% glutaraldehyde in 100 mM cacodylate buffer for 2 h, followed by 1% osmium tetroxide in 100 mM cacodylate buffer for 1 h. Specimens were then dehydrated in a graded series of alcohols/xylene, permeated with propylene oxide, and embedded in epoxy resin. Ultrathin sections of representative areas were stained with uranyl acetate and lead citrate and viewed with a JEM-1200EX electron microscope (Japan Electron Optics Laboratory).

### Axonal flow observation

The NTRK1 expression vector (RG213091; ORIGENE), the pPAmCherry-Mito Vector (TaKaRa Bio), and the pcDNA™6.2/C-EmGFP Vector (Invitrogen) were electroporated into cultured cells. For electroporation, NEPA21 (NEPAGENE) with platinum electrodes was used. The cultured colony was injected with fast green-dyed DNA solution using a sharp glass pipette, placed between the electrodes, and electroporated with voltage pulses (poring pulse: voltage 100 V, width 2.5 ms, interval 50 ms, two times; transfer pulse: voltage 20 V, width 50 ms, interval 50 ms, five times). The cultured cells were then allowed to develop in humidified incubators. Observations were made with an IX71 inverted research microscope (Olympus).

The time series of anterograde axonal flow was conducted by injection of Alexa Fluo-555 conjugated cholera toxin subunit B (Life Technologies) into the RGCR. Time-lapse analysis was performed immediately after the injection of cholera toxin B with a DeltaVision ELITE (CORNES Technologies).

### Electrophysiological recordings

Colonies were cultured on mixed cellulose ester filter papers (0.2-μm pore size; ADVANTEC) for 1 week. After removal of filter paper bearing colonies from the medium, suction was applied to the bottom of the filter to cause the colonies to attach firmly. Slices 200 μm thick were then cut vertically with a custom-made tissue chopper and fixed to the glass bottom of a recording chamber having a volume of 1.5 mL with a small amount of silicone grease (Dow Corning). All experiments were performed at physiological temperatures (35–37°C) using a ThermoClamp-1 (Automate Scientific). The chamber was continuously perfused at 1.5 mL/min with extracellular solution containing 120 mM NaCl, 3 mM KCl, 2.5 mM CaCl_2_, 1 mM MgCl_2_, 10 mM glucose, and 25 mM NaHCO_3_ equilibrated with 95%/5% O_2_/CO_2_ (pH 7.4). Chemicals were purchased from Sigma unless otherwise noted. Whole-cell patch-clamp recordings were made from the retinal ganglion cells located on the outer perimeter of the cultured colony. Recordings were performed with an Axopatch 200B amplifier (Molecular Devices) using pCLAMP 9.2 software (Molecular Devices). The slice preparations were visualised using an upright microscope (BX50WI; Olympus) equipped with DIC optics and a 60× water-immersion objective. The voltage or current trace was low-pass filtered (Bessel filter, corner frequency 10 kHz) and sampled at 20–50 kHz with a Digidata 1322A interface (Molecular Devices). Voltage-dependent Na^+^ currents were measured with leakage and capacitive current subtraction (P/-5 protocol) and were averaged over three trials. In some experiments, we added 1 μM tetrodotoxin (TTX) to the extracellular solution to block voltage-dependent Na^+^ channels. The recording pipettes (6–8 MΩ) were filled with an intracellular solution containing 120 mM K gluconate, 6 mM KCl, 2 mM NaCl, 1 mM CaCl_2_, 1 mM MgCl_2_, 5 mM EGTA, 10 mM HEPES, 4 mM Na_2_ATP, and 0.5 mM GTP (pH 7.2). In all experiments, Lucifer Yellow CH dilithium salt (LY; 0.05%) was added to the intracellular solutions to visualise the morphology of the recorded cells. Liquid junction potentials (−11 mV) were corrected. The average series resistance (Rs) was 29.4 ± 4.77 MΩ (n = 5). Rs was compensated by 40%. Data with Rs values of more than 35 MΩ were excluded from the analyses. The average membrane capacitance during the recordings was 7.20 ± 2.84 pF (n = 5). Current and voltage data were acquired using pCLAMP 9.2 software and saved on a custom-built personal computer (Physio-Tech). Analyses were performed with Clampfit 9.2 (Molecular Devices) and OriginPro 8J (OriginLab). Images of LY-filled cells were captured using a high-gain colour camera (HCC-600; Flovel) and saved using INFO.TV Plus software (Infocity). The images were adjusted for brightness and contrast and complemented by pasting in a part of another image obtained from a different depth of the slice preparation using Photoshop CS6 software (Adobe Systems). All data are presented as means ± SDs.

## Author Contributions

T.T. and T.Y. performed experiments, analysed and interpreted data, and wrote the manuscript. F.T. and S.W. designed and performed the electrophysiological recordings and contributed to the writing of the manuscripts. S.N. interpreted data. N.A. designed experiments and supervised the project.

## Supplementary Material

Supplementary Information Figure S1. S2. S3. S4. S5. S6. S7. S8. S9. Table S1. S2. S3.

Supplementary Information Video S1.

Supplementary Information Video S2.

Supplementary Information Video S3.
