# Supplementary material for: Generation of retinal ganglion cells with functional axons from human induced pluripotent stem cells
Source: Sci Rep. 2015 Feb 10;5:8344. doi: 10.1038/srep08344 (PMC4322369; doi:10.1038/srep08344)
Supplement: Supplementary Information — Figure S1. S2. S3. S4. S5. S6. S7. S8. S9. Table S1. S2. S3. [file srep08344-s1.doc]

**Generation of retinal ganglion cells with functional axons from human induced pluripotent stem cells**

Taku Tanaka^1^, Tadashi Yokoi^1^, Fuminobu Tamalu^2^, Shu-Ichi Watanabe^2^, Sachiko Nishina^1^ and Noriyuki Azuma^1^

**Figure captions**

**Figure S1.** Representative images of floating embryoid bodies with several optic vesicles (OVs) under different conditions. Optic vesicles were well organised under (a) 1% FBS and 0.5% Matrigel, (b) 5% FBS and 1.0% Matrigel, and (c) 10% FBS and 2.0% Matrigel. There were no significant differences in the development of OVs among these conditions.

**Figure S2.** Ideal timing of attachment of embryoid bodies and accompanying optic vesicles. (a) Attachment before D25 failed to yield axons, and many rosette-like immature structures appeared. (b) Attachment after D30 also showed insufficient attachment, and axons were not observed.

**Figure S3.** Additional of retinoic acid (0.5 µM) 3 days prior to attachment promoted axon elongation on D30. (a) Abundant axons grew from the three neighbouring clumps of cells when retinoic acid was added prior to attachment of embryoid bodies with optic vesicles. (b) Without retinoic acid supplementation, only very small axons were observed from the clumps.

**Figure S4.** Continuous supplementation with retinoic acid after attachment of embryoid bodies induced early elimination of axons. (a) Dishes were continuously supplemented with retinoic acid beginning from 3 days prior to attachment to D37, resulting in the loss of axons. (b) Dishes were supplemented with retinoic acid 3 days prior to attachment and then continuously supplemented with BDNF (100 ng/mL) after attachment instead of retinoic acid. The axons continued to grow.

**Figure S5.** Stereomicroscopic images of surviving axons until D50 during supplementation with BDNF (100 ng/mL). (a, b) Ganglion cells with axons generally survived at least until D40 (a), with maximum survival observed at approximately D50 (b) under the culture conditions used in the current experiments. The axons began to split from the dish at D52.

**Figure S6.** Immunohistochemistry on D35 for syntaxin, calbindin, and PKCα. Negative controls were stained only with secondary antibody. (a) Syntaxin, an amacrine cell marker, gradually increased following increased Brn3b expression, and low levels were detected by immunostaining on D34. (b) Calbindin, a horizontal cell marker, was not expressed on D35. (c) PKCα, a bipolar cell marker, was also not detected. (d) Negative staining with secondary antibody only was used to exclude non-specific staining.

**Figure S7.** Nissl bodies of retinal ganglion cells. (a) Retinal ganglion cells possessed characteristic Nissl bodies. Axons elongated from the retinal ganglion cells. (b) Small round cells in the rosette did not show any Nissl bodies.

**Figure S8.** Initial images taken from Video S1 of the time lapse analysis of axonal transport. Arrows indicate representative Alexa-Fluo-555-conjugated cholera toxin B with fast flow, and arrowheads indicate representative Alexa-Fluo-555-conjugated cholera toxin B with slow flow.

**Figure S9.** Initial images taken from Video S2 of the time lapse analysis of axonal transport. Arrowheads indicate representative Alexa-Fluo-555-conjugated cholera toxin B with slow flow.

**Table S1.** The effects of FBS and Matrigel concentrations on the formation of optic vesicles (OVs). The frequency of OV formation from embryoid bodies (EBs) was examined under different Matrigel and FBS. Twelve EBs were used to evaluate the formation of OVs on day 25 for each condition. The numbers in the columns show the percentages of EBs with OV-like structures.

**Table S2.** Primer list. The primer sets were used for real-time PCR analysis.

**Table S3.** Antibody list. The antibodies were used for immunohistochemistry.

**Video S1.** Time lapse analysis of axonal transport. A time series of axonal transport was also recorded by the injection of Alexa-Fluo-555-conjugated cholera toxin B into the retinal ganglion cell region. Fast and slow flows were obviously identified.

**Videos S2 and S3.** Inhibition of axonal transport by colchicine. Axonal transport of Alexa-Fluo-555-conjugated cholera toxin B (S2) was blocked immediately by an addition of 1 mM colchicine (S3).

**Figure S1.**


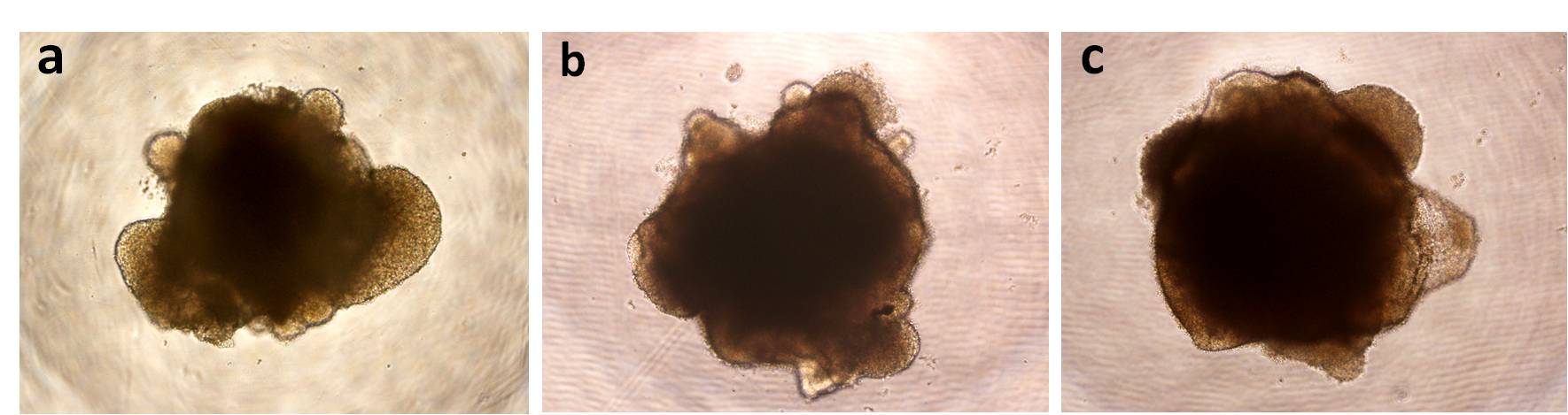


**Figure S2.**

**
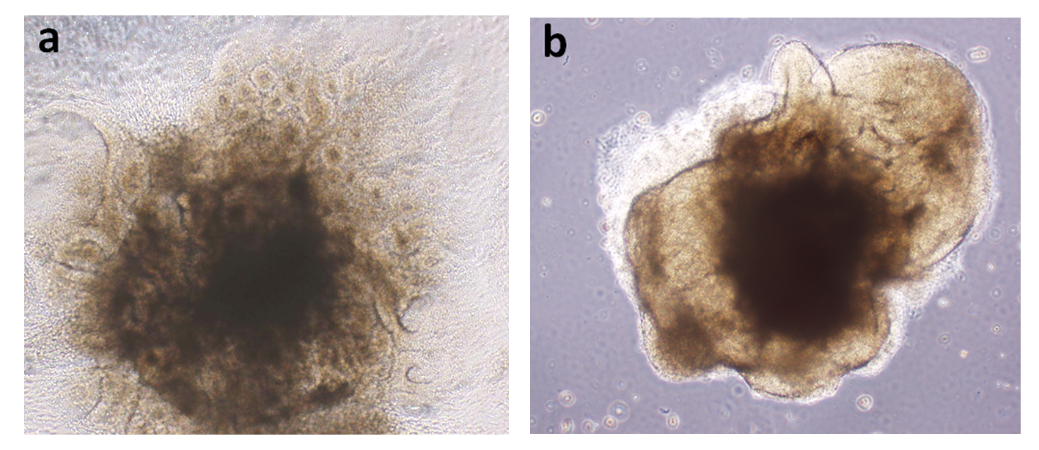
**

**Figure S3.**

**
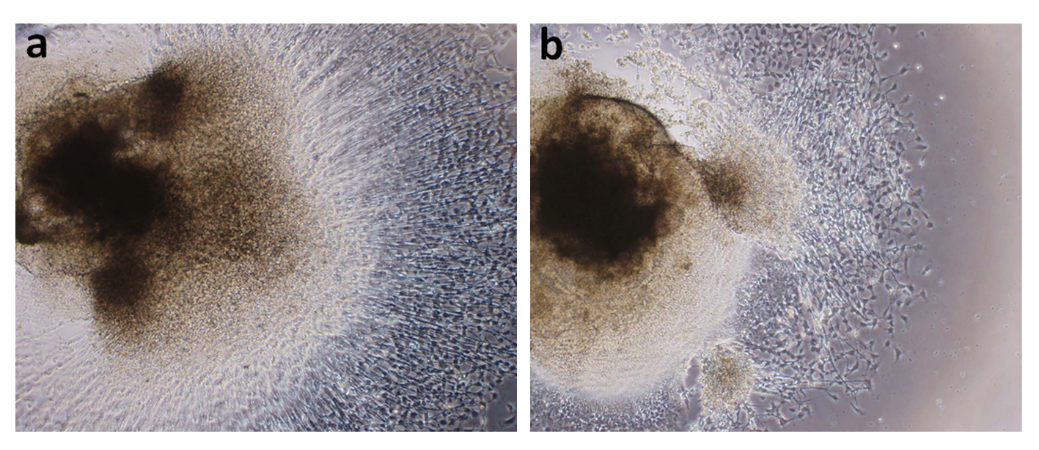
**

**Figure S4.**

**
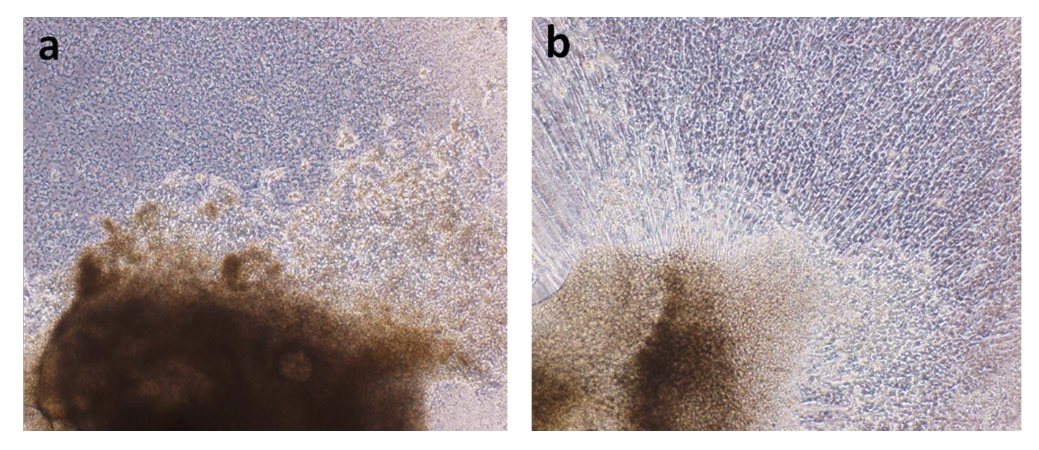
**

**Figure S5.**

**
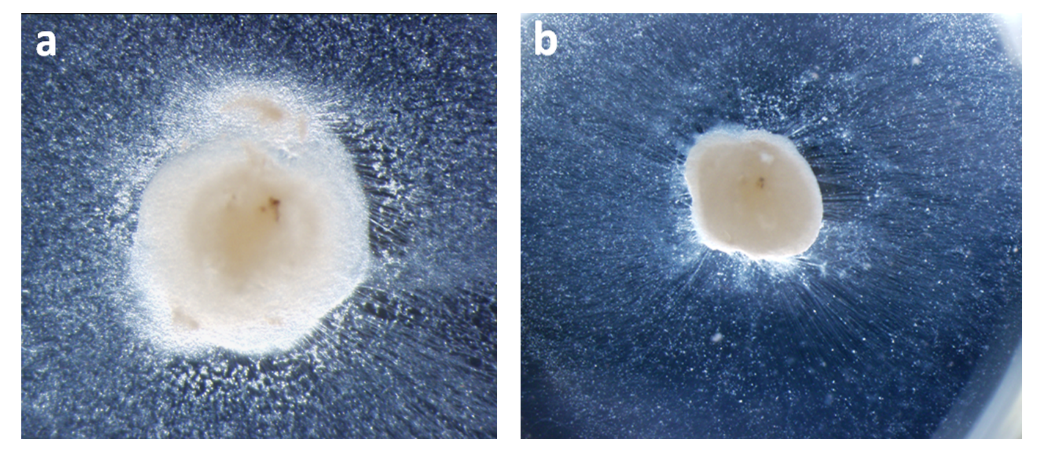
**

**Figure S6.**

**
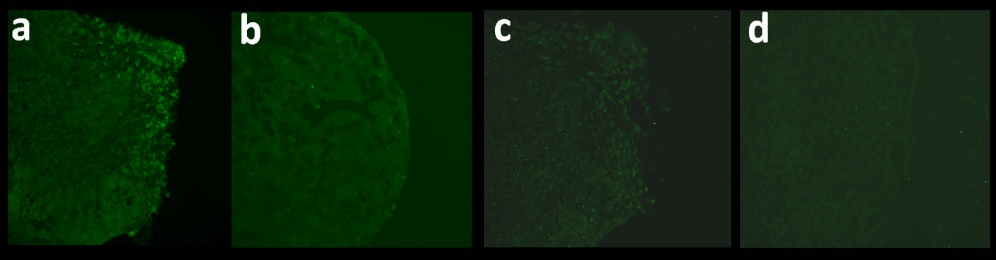
**

**Figure S7.**

**
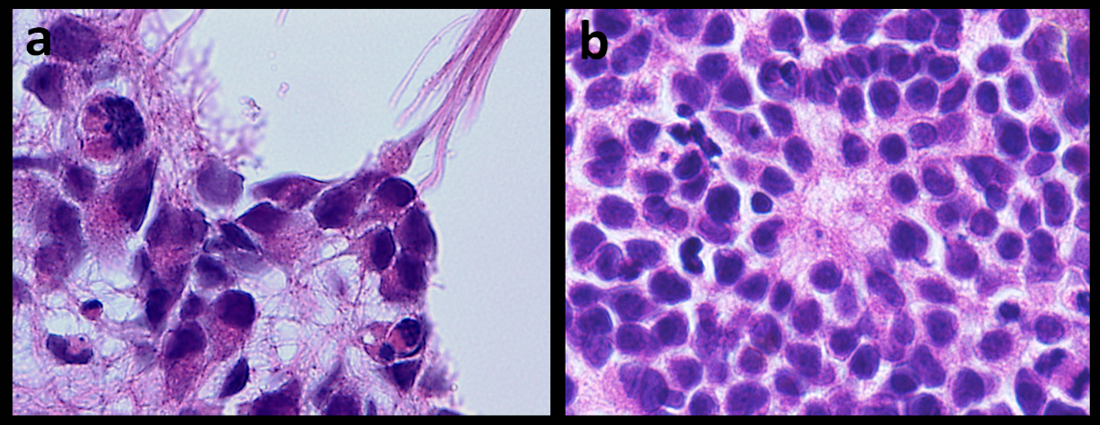
**

**Figure S8.**

**
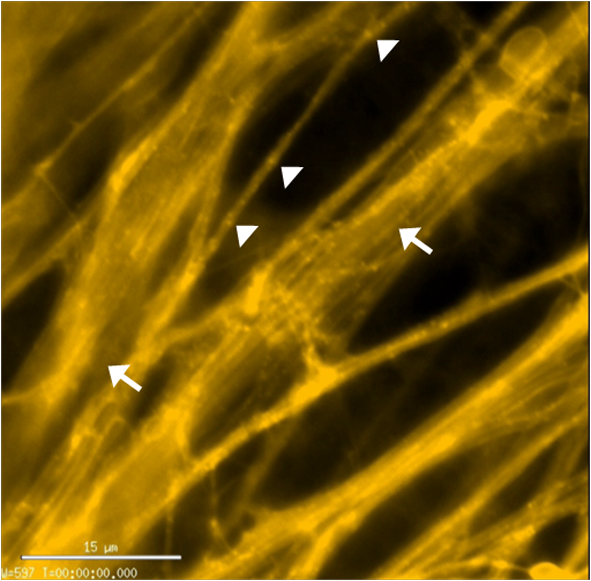
**

**Figure S9.**

**
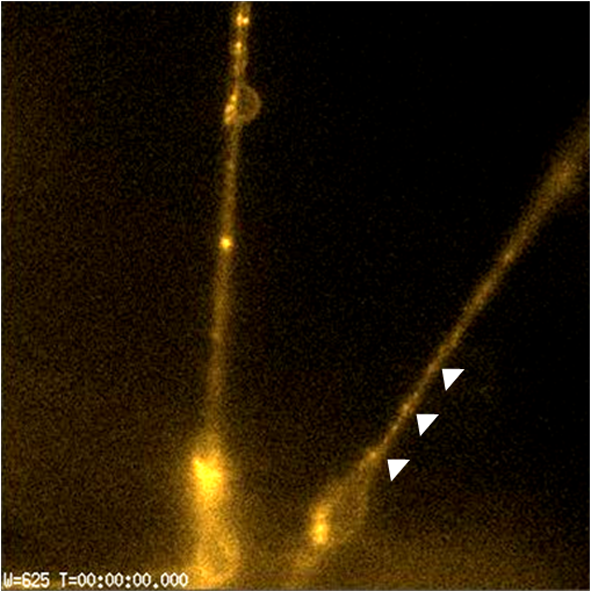
**

**Table S1.**

| FBS (%)  Matrigel (%) | 1 | 5 | 10 |
| --- | --- | --- | --- |
| 0.5 | 92% (11/12) | nd | 75% (9/12) |
| 1.0 | 83% (10/12) | 92% (11/12) | 83% (10/12) |
| 2.0 | 83% (10/12) | nd | 83% (10/12) |

nd: no data

**Table S2.**

|  | Gene | Accession No | Forward | Reverse |
| --- | --- | --- | --- | --- |
| 1 | *Hprt1* | NM_000194.2 | GGCAGTATAATCCAAAGATGGTCAA | GTCAAGGGCATATCCTACAACAAAC |
| 2 | *Rx* | NM_013435.2 | CCGTCCCTAAGCGTGCTTTC | ACTGGGAGCTTCACTAATTTGCTCA |
| 3 | *Pax6* | NM_000280.4 | TTTAAAGATCCTGGAGGTGGACATA | GCTCAGGTGCTCGGGTTCTA |
| 4 | *Chx10* | NM_182894.2 | AACCCAATCTGGCTGGTAAATGA | CAGCAGGCCCTTAATGCGTA |
| 5 | *Six3* | NM_005413.3 | GCAGAAGACGCATTGCTTCAA | GTTCGCGTTTCTTGCTGGG |
| 6 | *Brn3b* | NM_004575.2 | TGACACATGAGCGCTCTCACTTAC | ACCAAGTGGCAAATGCACCTA |
| 7 | *Crx* | NM_000554.4 | ACCCTGATCTCTAGAGCCCACAA | CTTAATGTCCCAGAACCCAGCA |
| 8 | *Syntaxin* | NM_004603.3 | TAAAGAGCATCGAGCAGTCCA | GACATGACCTCCACAAACTTTCT |
| 9 | *Calbindin* | NM_004929.2 | TCCAGGGAATCAAAATGTGTGG | GCACAGATCCTTCAGTAAAGCA |
| 10 | *PKCα* | NM_002737.2 | ACAACCTTCCAACAACCTTGAC | CCTTCCTGTCGGCAAGCAT |
| 11 | *Math5* | NM_145178.3 | CCCTAAATTTGGGCAAGTGAAGA | CAAAGCAACTCACGTGCAATC |
| 12 | *Mitf* | NM_198159.2 | AGAGTCTGAAGCAAGAGCACTG | TGCGGTCATTTATGTTAAATCTTC |
| 13 | *Tuj1* | NM_006086.3 | GGCCAAGGGTCACTACACG | GCAGTCGCAGTTTTCACACTC |
| 14 | *Islet1* | NM_002202.2 | GCGGAGTGTAATCAGTATTTGGA | GCATTTGATCCCGTACAACCT |
| 15 | *Sncg* | NM_003087.2 | TGAGCAGCGTCAACACTGTG | GAGGTGACCGCGATGTTCTC |
| 16 | *Tau* | NM_001123066.3 | CCAAGTGTGGCTCATTAGGCA | CCAATCTTCGACTGGACTCTGT |
| 17 | *NFL* | NM_006158.4 | TCAACGTGAAGATGGCTTTGGATA | AAGACCTGGGAGCTCTGGGAGTA |
| 18 | *NFM* | NM_005382.2 | ACAACCACGACCTCAGCAGCTA | ATGACGAGCCATTTCCCACTTT |
| 19 | *NFH* | NM_021076.3 | CAGCTGCGAGAATACCAGGAC | CACCTTTATGTGAGTGGACACAGAG |

**Table S3.**

|  | Gene Product | Supplier | Product Number | Dilution |
| --- | --- | --- | --- | --- |
| 1 | RX | Thermo Fisher Scientific | PA5-11477 | 1:25 |
| 2 | PAX6 | Covance | PRB-278P | 1:50 |
| 3 | BRN3B | Santa Cruz | sc-31989 | 1:25 |
| 4 | CRX | Abnova | H00001406-M02 | 1:50 |
| 5 | MATH5 | Millipore | AB5694 | 1:50 |
| 6 | ISLET1 | Abcam | ab20670 | 1:50 |
| 7 | SNCG | GeneTex | GTX110483 | 1:50 |
| 8 | TUJ1 | Sigma | T-5076 | 1:200 |
| 9 | TAU | Santa Cruz | sc-5587 | 1:100 |
| 10 | NFL | Cell Signaling Technology | #8024S | 1:100 |
| 11 | NFH | Abcam | ab8135 | 1:100 |
